# A novel CT-based radiomics approach for kidney function evaluation in ADPKD: a pilot study

**DOI:** 10.1093/ckj/sfaf264

**Published:** 2025-09-04

**Authors:** Luca Calvaruso, Pierluigi Fulignati, Luigi Larosa, Huong Elena Tran, Claudio Votta, Carla Cipri, Luigi Natale, Viola D'Ambrosio, Giulia Condello, Pietro Manuel Ferraro, Francesco Pesce, Luca Boldrini, Giuseppe Grandaliano

**Affiliations:** Nephrology, Dialysis and Transplantation Unit, Department of Medical and Surgical Sciences, Fondazione Policlinico Universitario A. Gemelli IRCCS, Rome, Italy; Nephrology, Dialysis and Transplantation Unit, Department of Medical and Surgical Sciences, Fondazione Policlinico Universitario A. Gemelli IRCCS, Rome, Italy; Department of Diagnostic Imaging and Radiation Oncology, Fondazione Policlinico Universitario “A. Gemelli” IRCCS, Rome, Italy; Department of Diagnostic Imaging and Radiation Oncology, Fondazione Policlinico Universitario “A. Gemelli” IRCCS, Rome, Italy; Department of Diagnostic Imaging and Radiation Oncology, Fondazione Policlinico Universitario “A. Gemelli” IRCCS, Rome, Italy; Department of Diagnostic Imaging and Radiation Oncology, Fondazione Policlinico Universitario “A. Gemelli” IRCCS, Rome, Italy; Department of Diagnostic Imaging and Radiation Oncology, Fondazione Policlinico Universitario “A. Gemelli” IRCCS, Rome, Italy; Department of Translational Medicine and Surgery, Università Cattolica del Sacro Cuore, Rome, Italy; Nephrology, Dialysis and Transplantation Unit, Department of Medical and Surgical Sciences, Fondazione Policlinico Universitario A. Gemelli IRCCS, Rome, Italy; Nephrology, Dialysis and Transplantation Unit, Department of Medical and Surgical Sciences, Fondazione Policlinico Universitario A. Gemelli IRCCS, Rome, Italy; Section of Nephrology, Department of Medicine, Università degli Studi di Verona, Verona, Italy; Department of Translational Medicine and Surgery, Università Cattolica del Sacro Cuore, Rome, Italy; Nephrology Unit, Ospedale Isola Tiberina-Gemelli Isola, Rome, Italy; Department of Diagnostic Imaging and Radiation Oncology, Fondazione Policlinico Universitario “A. Gemelli” IRCCS, Rome, Italy; Nephrology, Dialysis and Transplantation Unit, Department of Medical and Surgical Sciences, Fondazione Policlinico Universitario A. Gemelli IRCCS, Rome, Italy; Department of Translational Medicine and Surgery, Università Cattolica del Sacro Cuore, Rome, Italy

**Keywords:** autosomal dominant polycystic kidney disease, imaging, radiomics, renal function

## Abstract

**Background:**

Management of autosomal dominant polycystic kidney disease (ADPKD) might take advantage of the use of new tools to predict risk of progression towards end-stage kidney disease (ESKD). The aim of this study is to explore the potential of radiomic features obtained from computed tomography (CT) scans for the prediction of kidney function decline over time of ADPKD patients.

**Methods:**

We retrospectively selected a cohort of 58 ADPKD patients who routinely underwent CT scan for total kidney volume (TKV) assessment from February 2020 to March 2021. An expert radiologist generated a region-of-interest segmentation for cystic kidneys from which we extracted 217 radiomic features. In a subgroup of 51 patients with at least three serum creatinine measurements, on the basis of estimated glomerular filtration rate we identified 26 rapid progressors to ESKD (>3 mL/min/1.73 m^2^/year), and we developed a radiomic model to discriminate rapid from non-rapid progressors. Area under the curve (AUC) of the receiver operating characteristic (ROC) and sensitivity were employed to evaluate models’ performance.

**Results:**

The most statistically significant radiomic feature (*F_cm.corr*) (*P*-value = .04) associated with rapid progression showed an AUC (95% confidence interval) of 0.78 (0.65–0.90) and a sensitivity of 0.92 (0.78–0.98). On the contrary, the logistic regression model based on the height-adjusted TKV (ht-TKV) presented a lower AUC (95% confidence interval) of 0.65 (0.49–0.80), with a sensitivity 0.62 (0.42–0.78).

**Conclusions:**

We developed a model based on the radiomic feature *F_cm.corr* that was able to discriminate rapid progressors. Further validation studies on larger and external cohort are warranted to corroborate our findings and to confirm the role of radiomics in ADPKD management.

## Graphical Abstract

**Graphical Abstract:**
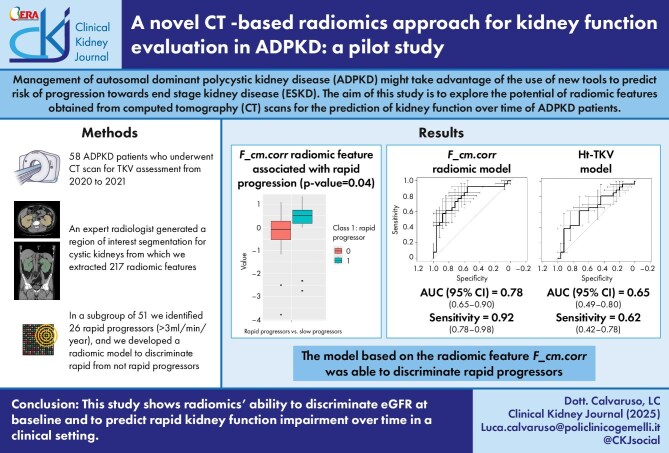

KEY LEARNING POINTS **What was known:**

- Autosomal dominant polycystic kidney disease (ADPKD) is a systemic disease primarily characterized by progressive development of multiple bilateral renal cysts and kidney function impairment that eventually leads to end-stage kidney disease (ESKD) at a median age of 56 years in approximately 70% of patients.
- Rapid decline in estimated glomerular filtration rate as well as high rate of total kidney volume (TKV) growth per year are by now acknowledged as predictive tools of rapid disease progression.
- Radiomics consists of high-throughput extraction of quantitative image features converted into high-dimensional data and has been employed in risk prediction of many oncological diseases but very few studies have focused so far on radiomic features originating from non-neoplastic kidney tissue to create clinical models of predictive significance.

**This study adds:**

- In our cohort, comprehensive of 58 ADPKD patients who routinely underwent computed tomography scan for total kidney volume assessment, we identified a radiomic feature (*F_cm.corr*) significantly associated with rapid progression.
- A model based on the radiomic feature *F_cm.corr* was able to better discriminate rapid progressors compared with standard than TKV.

**Potential impact:**

- This is among the first studies aiming to investigate radiomics’ potential ability to predict faster rapid kidney function impairment over time in a clinical setting.
- This could represent a groundbreaking approach to improve the stratification of patients’ risk of progression towards ESKD and to better treat specific groups of patients with novel disease-modifying therapies.
- To confirm the supporting role of radiomics to clinical decisions in the management of ADPKD, further studies should implement a model with a larger cohort and external validation.

## INTRODUCTION

Autosomal dominant polycystic kidney disease (ADPKD) is the most prevalent monogenic hereditary kidney disease. It is a systemic disease primarily characterized by progressive development of multiple bilateral renal cysts and kidney function impairment that in approximately 70% of patients eventually leads to end-stage kidney disease (ESKD) at a median age of 56 years [1, 2]. In the context of ADPKD pathogenesis, the histological scenario is markedly influenced by fibrosis and inflammation [3, 4].

In recent years, great effort has been made to find a biomarker that predicts kidney function decline and identifies individuals at high risk of progression towards ESKD that could benefit from disease-modifying therapy. Rapid estimated glomerular filtration rate (eGFR) decline as well as high rate of total kidney volume (TKV) growth per year are acknowledged as predictive tools of rapid disease progression [5]. TKV, in particular, was formally recognized, both by the Food and Drug Administratin and the European Medicines Agency, as a prognostic imaging biomarker for kidney function decline. Mayo Imaging Classification (MIC), the most valuable method for risk prediction, is of widespread use in the nephrology community and derives from either computed tomography (CT) or magnetic resonance imaging (MRI) imaging data [6]. Lately, radiomics consisting of a high-throughput extraction of quantitative image features and conversion of medical data into high-dimensional data has successfully been employed in prediction and staging of many oncological diseases [7, 8]. Few scientific studies have focused so far on radiomic features originating from non-neoplastic kidney tissue in the perspective of creating clinical models of predictive significance.

The aim of the present study is to explore the ability of radiomics to predict kidney function as well as the rate of its decline over time in ADPKD.

## MATERIALS AND METHODS

### Study population

We retrospectively selected a cohort of ADPKD patients afferent to our ADPKD outpatient clinic at Policlinico A. Gemelli, IRCCS in Rome from February 2020 to March 2021. Our inclusion criteria were: (i) patients who had a radiological diagnosis of ADPKD either with ultrasound or CT, and (ii) patients who underwent CT scan to define TKV and to apply the MIC as a risk-stratification tool [6]. All patients on tolvaptan at the time of the study had initiated the treatment prior to the defined observation period of the study.

The following clinical information were retrospectively collected through electronic medical records at the time of CT risk assessment: age, gender, serum creatinine, eGFR calculated with Chronic Kidney Disease Epidemiology Collaboration (CKD-EPI), CKD stage, baseline TKV adjusted for height (ht-TKV) calculated by means of CT and MIC. The present study had clinical committee approval by the Institutional Review Board of Fondazione Policlinico A. Gemelli IRCCS (notification—UCT2: ID 5823).

### Image acquisition

CT was performed with 64-row spiral CT scanner using the following parameters: 1.5 mm slice thickness, 1.25 mm slice interval, 120 kV tube voltages, automated tube current modulation (200–700 mA), pitch of 0.969 mm/rot, 0.9 s rotation time. Modulation of tube current (mA) and voltage (kV) allowed dose saving, especially in young patients, but image quality was not compromised at the same time.

CT scans covered the entire abdominal cavity in order to fully include both kidneys, and no contrast medium was used.

### Image segmentation

The first post-processing evaluation was performed on a GE Healthcare workstation (version 4.7), using the reformat tool for measurement of each kidney volume (right and left volumes) and consequently of TKV (sum of right and left volumes). Manual segmentation of the region of interest (ROI) including the whole kidney parenchyma, was subsequently performed using 3D Slicer version 4.13 (http://www.slicer.org), by a radiologist with specific expertise in kidney imaging.

**Figure 1:**
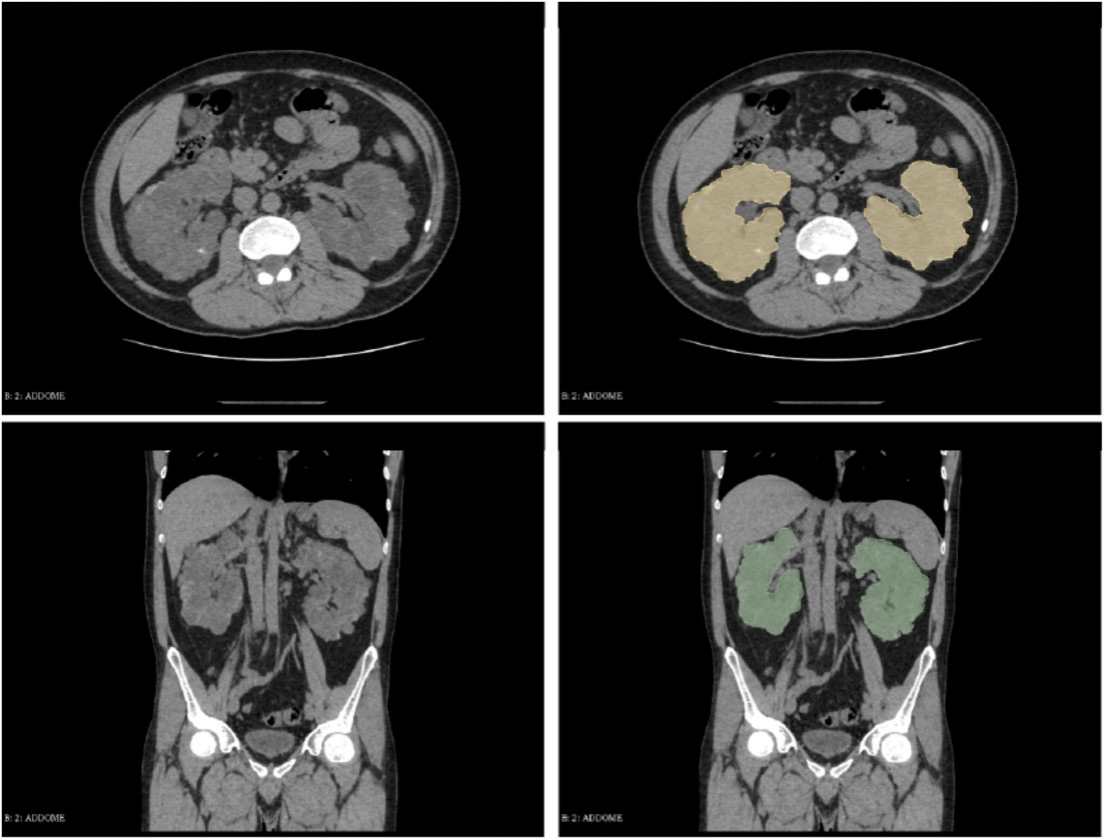
Axial and coronal CT images without intravenous contrast injection, before and after kidney segmentation. Note that the hilum of both kidneys was not included in the segmentation.

Segmentation was carried out slice-by-slice in axial CT scans. Vascular structures of renal hilum and renal pelvis were excluded from the segmentation. Two segmentations, one for the right kidney parenchyma and the other for the left one, were performed. The union Boolean operator was applied to these two segmentations to obtain the ROI consisting of the whole kidney parenchyma, which was then used for the radiomic analysis (Fig. 1).

### Radiomic feature extraction

All radiomic analyses were performed by using RStudio (R version 4.1.3). Radiomic features were extracted from each ROI using MODDICOM, an open-source R library developed by the Radiomics Research Core facility of the Policlinic A. Gemelli, IRCCS, Rome [9].

Calculation of the radiomic features in 2D and 2.5D was proved to be fully compliant with the recommendations of the Image Biomarker Standardization Initiative (IBSI) [10].

Different families of radiomic features were extracted. Morphological features describe geometric characteristics of the ROI. Intensity-based statistical features represent statistical global measures of the grey-level histogram of the ROI. Textural features implement instead local measures of the distribution of the grey levels in the ROI. Z-score normalization was applied to each radiomic feature across all patients before further analysis.

### Baseline eGFR-based kidney function

Our cohort of patients was stratified into two groups according to the eGFR-based kidney function at baseline with a cut-off of 60 mL/min/1.73 m^2^: late CKD <60 mL/min/1.73 m^2^ or early CKD ≥60 mL/min/1.73 m^2^.

We built and compared three different logistic regression models to perform the binary classification late CKD vs early CKD: a model based on ht-TKV and age, a model based on a selected radiomic feature and age, and a third model based on ht-TKV and the selected radiomic feature. Radiomic features’ selection was conducted to identify one radiomic feature for model development while avoiding overfitting. The selection of only one radiomic feature was made according to the one-to-ten rule (related to the number of events per variable) for the logistic regression models [11], considering that the selected feature was also used for the development of the combined model with the variables ht-TKV and age. To this end, first, a univariate analysis with the Wilcoxon–Mann–Whitney test was performed to identify radiomic features presenting a statistically significance difference between the two classes of eGFR-based kidney function (late CKD <60 mL/min/1.73 m^2^ or early CKD ≥60 mL/min/1.73 m^2^). The Benjamin–Hochberg procedure was adopted to correct for multiple testing comparisons with a significance level of .05. Then, a correlation analysis of the features with the lowest *P*-value was carried out by means of the Pearson coefficient identifying the feature showing the lowest mean absolute correlation with the others.

The three models were built by fitting a logistic regression to the data. Model calibration was evaluated by performing a likelihood-based statistical test with a significance level of .05, comparing the probabilities estimated by the models with the observed outcomes [3, 12].

Model discrimination was assessed by calculating the area under the curve (AUC) of the receiver operating characteristic (ROC) curve. Stratified bootstrap resampling was applied with 2000 iterations to obtain the 95% confidence interval (CI) for the AUCs. The DeLong method was used to compare the discriminative ability of the three developed models. The significance level was set to .05.

Classification metrics including accuracy, sensitivity and specificity were computed by setting the probability threshold according to the Youden's index method. Sensitivity and specificity referred to the patients affected by late CKD and those with early CKD, respectively. The Jeffreys method suitable for small sample sizes was used to compute the 95% CI for the classification metrics [4].

The internal validation of the models was conducted by performing a 3-fold cross-validation (CV) repeated five times, and computing mean and standard deviations of the model performance metrics over the CV repetitions.

Continuous clinical variables of the patient populations were reported as mean (standard deviation) or median (interquartile range) and categorical clinical variables were described as number (%). Continuous clinical variables were reported as mean and 95% CI if normally distributed, and medians and Q1–Q3 if not. Discrete clinical variables were reported as percentages. Groups stratified with a cut-off of 60 mL/min/1.73 m^2^ were compared with a *t*-test; Wilcoxon or chi-squared tests were used when appropriate.

### Rapid progression to ESKD

We developed a preliminary radiomic model to discriminate rapid from non-rapid progressors to ESKD. For this analysis we considered a subgroup of patients who had at least three serum creatinine measurements with a minimum follow up of 6 months and an average total follow-up period of 18.6 months: timing for blood measurements was in line with the indication from the KDIGO 2024 Clinical Practice Guideline for CKD stages and/or to specific treatment (tolvaptan) safety monitoring indications [13]. Specifically, the average time interval between creatinine observation was 4.61 months; the minimum follow-up interval was 1 month which is the usual time interval for patients undergoing tolvaptan therapy adopted in order to strictly monitor kidney function and to exclude hepato-toxicity. The kidney function slope of eGFR measurements for each patient was estimated by fitting a linear regression model. Patients were subdivided in rapid and non-rapid progressors to ESKD by setting a threshold of 3 mL/min/1.73 m^2^/year [5].

Radiomic features’ selection was implemented following the same steps performed for the radiomic analysis of late CKD vs early CKD in the previous section. A radiomic model was then developed with the selected radiomic feature by fitting a logistic regression model, and compared with a logistic regression model built using the ht-TKV as the only input variable.

The AUC of the ROC curves was computed to assess the discrimination's ability of the models. The 95% CI for the AUC was obtained by means of 2000 iterations of stratified bootstrap resampling. Accuracy, sensitivity and specificity were computed by selecting the probability threshold as the Youden's index, with 95% CIs calculated according to the Jeffreys method. Sensitivity and specificity referred to rapid progressors and non-rapid progressors, respectively.

Internal validation of the radiomic model was performed by means of bootstrap resampling with 1000 replicates of the dataset and logistic regression models. The average values of the bootstrapped model coefficients were used to build an ‘average’ model, for which we assessed the AUC of the ROC curve and the classification metrics.

## RESULTS

### Clinical characteristics

**Figure 2:**
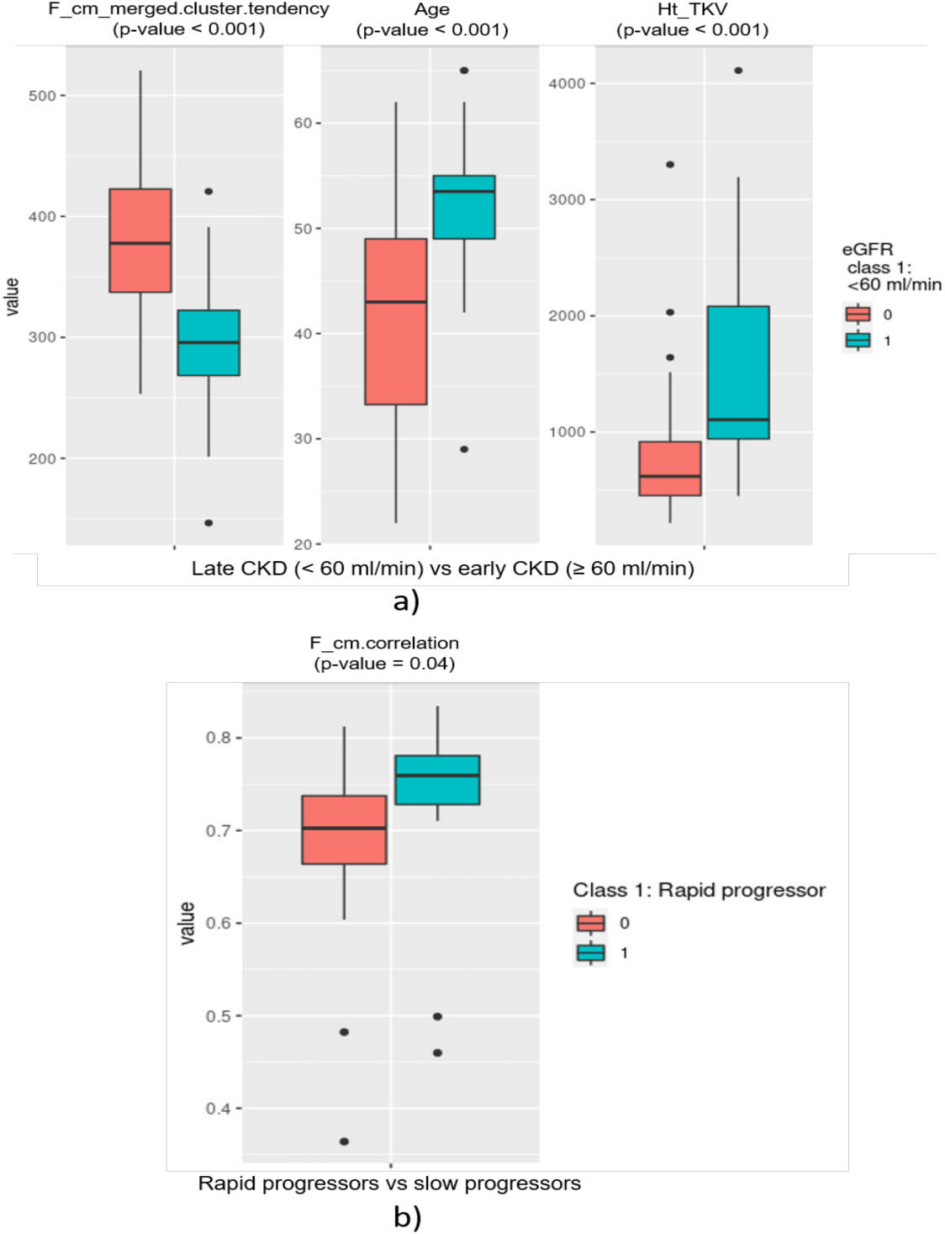
(**a**) Boxplot of the selected radiomic feature and the investigated clinical variable (ht-TKV) showing the discrimination between class 0 (eGFR ≥60 mL/min/1.73 m^2^) and class 1 (eGFR <60 mL/min/1.73 m^2^). (**b**) Boxplot of the selected radiomic feature showing the discrimination between rapid progressor patients (>3 mL/min/1.73 m^2^/year slope) from non-rapid progressor (<3 mL/min/1.73 m^2^/year slope). Box represents the interquartile range (IQR), the line inside the box indicates the median, and the whiskers extend to 1.5 times the IQR from the first and third quartiles. Any points beyond this range are plotted as outliers.

Our cohort included 58 patients affected by ADPKD. The two groups of patients stratified by eGFR were well balanced, including 30 early CKD patients with eGFR ≥60 mL/min/1.73 m^2^ and 28 late CKD patients with an eGFR <60 mL/min/1.73 m^2^ (Table 1). Age was higher among patients with eGFR <60 mL/min/1.73 m^2^: 52.4 vs 41.4 years (Fig. 2a). Fifteen patients were already on treatment with tolvaptan at the time of the study: among them, 13 had eGFR <60 mL/min/1.73 m^2^ and only 2 had an eGFR ≥60 mL/min/1.73 m^2^. As expected, median ht-TKV was higher (1061 mL) among patients with lower eGFR vs among patients with eGFR ≥60 mL/min/1.73 m^2^ (636 mL). For age, eGFR, ht-TKV there were significant differences between the two study groups (*P*-value <.001).

**Table 1:** Descriptive table of the study groups divided by eGFR and by rapid/non rapid CKD progression.

Clinical features	Overall	eGFR <60 mL/min/1.73 m^2^	eGFR ≥60 mL/min/1.73 m^2^	*P*-value	Rapid progressor	Non-rapid progressor	*P*-value
Patients, number	58	28	30		26	25	
Male sex, *n* (%)	26 (45)	14 (50)	12 (40)		12 (46.1)	11 (42.3)	
Age, mean ± SD	46.9 ± 10.8	52.4 ± 10.8	41.4 ± 10.6	<.001	49.1 ± 9.1	44.9 ± 12.3	.47
Patients on tolvaptan treatment	15	13	2		11	4	
eGFR (CKD-EPI, mL/min/1.73 m^2^), mean ± SD	62 ± 28.8	37 ± 28.8	84.8 ± 28	<.001	52 ± 26.7	67 ± 28.8	.06
ht-TKV, median (IQR)	917 (561–1370)	1061 (521–1251)	636 (569–1364)	<.001	1371,9 (735–2030)	1050,2 (555–1241)	.06
MIC, *n* (%)							
1A/1B	13 (22.4)	3 (10.7)	10 (33.3)	.01	4 (15.4)	5 (20)	.71
1C	27 (46.5)	16 (57.1)	11 (36.6)		12 (46.1)	13 (52)	
1D/E	18 (31.1)	9 (32.1)	9 (30)		10 (38.5)	7 (28)	

SD, standard deviation.

Fifty-eight ROIs, one per patient, were obtained from the segmentation step and 217 radiomic features were extracted from each ROI that included the kidney parenchyma.

### Baseline eGFR-based kidney function

The univariate analysis yielded 84 statistically significant features able to differentiate the two classes of eGFR-based kidney function, late and early CKD, respectively (<60 or ≥60 mL/min/1.73 m^2^), with six features presenting the lowest *P*-value (.0002). The correlation analysis with a threshold of 0.4 for the Pearson coefficient selected the cluster tendency computed from the co-occurrence matrix (*F_cm_merged.clust.tend*) as single radiomic feature for model development. As shown in Fig. 2a, we found that *F_cm_merged.clust.tend*, age as well as ht-TKV were able to robustly discriminate between eGFR classes. The boxplots reveal that the median value of the variable differs significantly between the two groups and that one group tends to have higher/lower values than the other.

**Figure 3:**
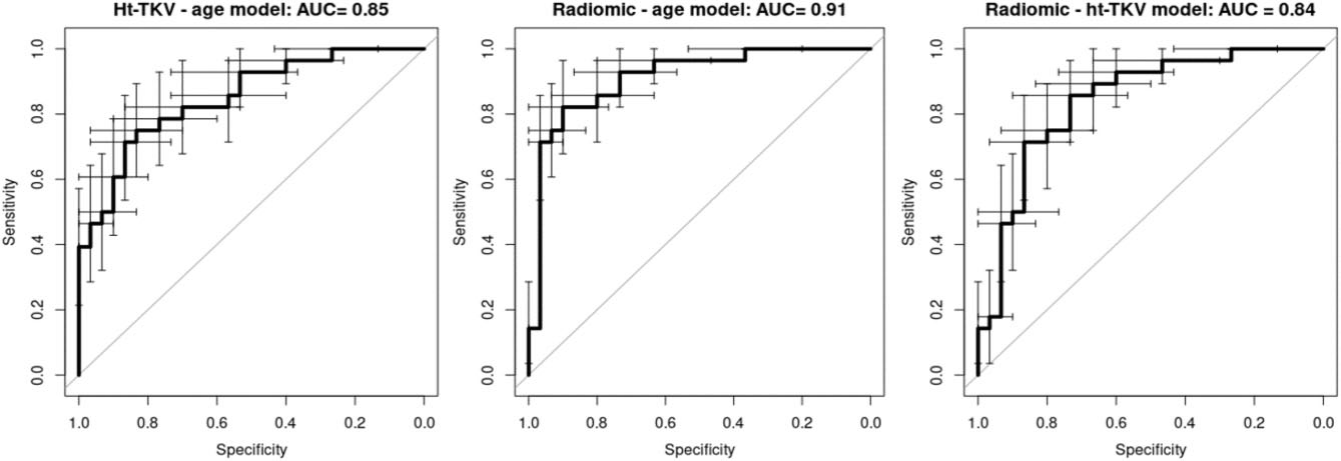
ROC curves from the logistic regression models (respectively, ht-TKV-age—based on ht-TKV and age, radiomic-age based on the radiomic feature *F_cm_merged.clust.tend* and age, and radiomic-ht-TKV model—based on ht-TKV and *F_cm_merged.clust.tend*) developed to differentiate the two classes of eGFR-based kidney function, late and early CKD, respectively (<60 or ≥60 mL/min/1.73 m^2^). The error bars indicate the 95% CI for sensitivity and specificity.

The three developed logistic regression models were calibrated with no statistically significant differences between model predicted probabilities and observed prevalence (*P*-values of .61, .60 and .56 for the ht-TKV-age, radiomic-age and radiomic-ht-TKV models, respectively). The ht-TKV-age, radiomic-age and radiomic-ht-TKV models presented, respectively, an AUC (95% CI) of 0.85 (0.75–0.95), 0.91 (0.83–0.99) and 0.84 (0.74–0.94) (Fig. 3), confirmed by the CV. No statistically significant difference in the discriminative ability was found between the radiomic-age and ht-TKV-age models (*P*-value = .35), or between the radiomic-age and radiomic-ht-TKV models (*P*-value = .30), or between the ht-TKV-age and radiomic-ht-TKV models (*P*-value = .91). Although a moderate Pearson correlation coefficient (–0.57) was found between the radiomic feature and ht-TKV, the radiomic feature alone did not represent a surrogate of ht-TKV since the discriminative ability of the combined model including both variables was statistically significantly different from that of the ht-TKV-only model (*P*-value <.01).

Table 2 reports classification metrics for the three models obtained during model fitting and after CV. The probability thresholds used to compute these metrics were 0.52 for the ht-TKV-age model, 0.50 for the radiomic-age model and 0.46 for the radiomic-ht-TKV model. The accuracy was higher for the radiomic-age model after model fitting (0.79, 0.86 and 0.79 for the ht-TKV-age, radiomic-age and radiomic-ht-TKV models, respectively. These results were confirmed by the CV with a mean value (standard deviation) over the CV iterations of 0.74, 0.82 and 0.77 for the ht-TKV-age, radiomic-age and radiomic-ht-TKV models, respectively. Thus, the radiomic-age model showed better discriminative ability and generalizability compared with the other models: it achieved 0.82 (0.65–0.93) for the sensitivity and 0.90 (0.76–0.97) for the specificity in the model fitting setting, and 0.79 (0.09) for the sensitivity and 0.86 (0.14) for the specificity in the CV setting.

**Table 2:** Classification metrics of the developed ht-TKV-age, radiomic-age (based on age and *F_cm_merged.clust.tend*), radiomic-ht-TKV (based on ht-TKV and *F_cm_merged.clust.tend*) models during model fitting and repeated 3-fold CV developed to differentiate the two classes of eGFR-based kidney function, late and early CKD, respectively (<60 or ≥60 mL/min/1.73 m^2^).

		Model fitting		
	AUC	Accuracy	Sensitivity	Specificity
ht-TKV-age	0.85 (0.75–0.95)	0.79 (0.68–0.88)	0.75 (0.57–0.88)	0.83 (0.67–0.93)
Radiomic-age	0.91 (0.83–0.99)	0.86 (0.76–0.93)	0.82 (0.65–0.93)	0.90 (0.76–0.97)
Radiomic-ht-TKV	0.84 (0.74–0.94)	0.79 (0.68–0.88)	0.86 (0.70–0.95)	0.73 (0.56–0.87)
		CV		
ht-TKV-age	0.83 (0.07)	0.74 (0.10)	0.72 (0.15)	0.78 (0.18)
Radiomic-age	0.90 (0.07)	0.82 (0.07)	0.79 (0.09)	0.86 (0.14)
Radiomic-ht-TKV	0.82 (0.07)	0.77 (0.08)	0.74(0.12)	0.79 (0.10)

Classification metrics are reported with their 95% CIs for model fitting, and with their standard deviations for the repeated 3-fold CV.

### Rapid progression to ESKD

In a subgroup of 51 patients who had throughout the follow-up at least three serum creatinine measurements, the estimate of kidney function slope resulted in the classification of 26 rapid progressors and 25 non-rapid progressors. Among rapid progressor patients 11 patients were on tolvaptan at the time of the study.

Notably, no statistical significance (*P*-value = .71) was found when we looked at the correlation between MIC classes, among rapid and non-rapid progressor patients (Table 1).

From the univariate analysis, two textural radiomic features (computed from the co-occurrence matrix using two different aggregation methods) demonstrated statistical significance: ‘*F_cm.corr*’ and ‘*F_cm_merged.corr*’. The correlation analysis between the two features revealed a strong correlation, with a Pearson correlation coefficient close to 1, leading to the selection for the radiomic model development of the sole ‘*F_cm.corr*’ which significantly increased among rapid progressor group (*P*-value = .04) (Fig. 2b). On the contrary no statistically significant difference was found for the ht-TKV (*P*-value = .06).

**Figure 4:**
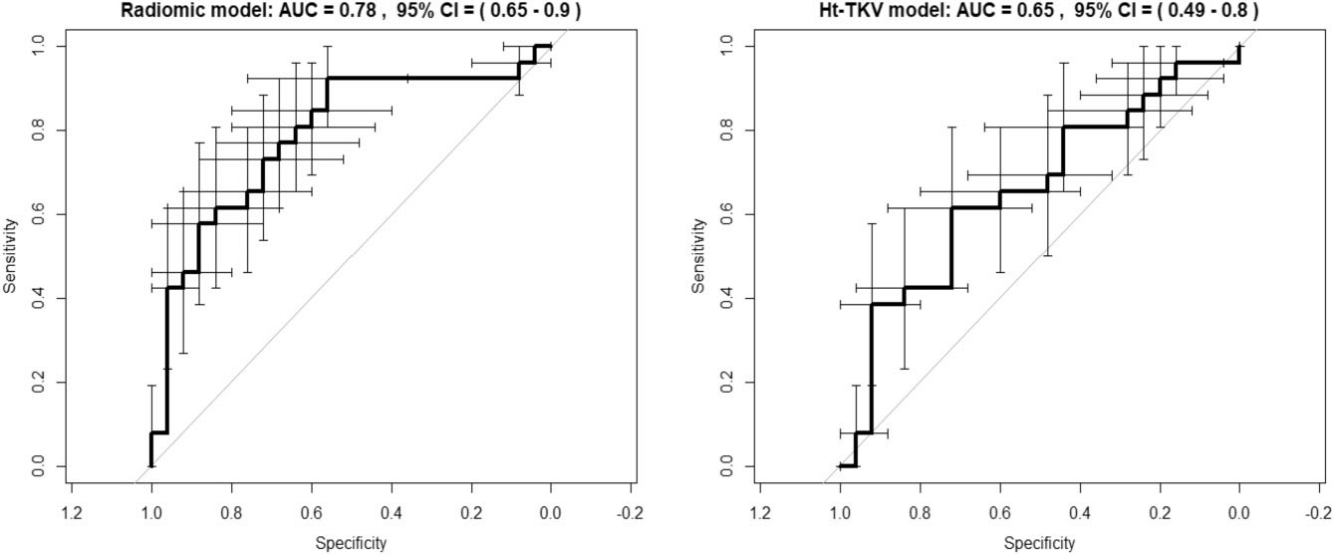
Comparison of the ROC curves from the model based on the radiomic feature *F_cm.corr* and the model based on the ht-TKV to discriminate between rapid and non-rapid progression towards ESKD. The error bars indicate the 95% CI for sensitivity and specificity.

The logistic regression model based on the selected feature presented an AUC (95% CI) of 0.78 (0.65–0.90) on the ROC curve. On the contrary, the logistic regression model based on the ht-TKV presented a lower AUC (95% CI) of 0.65 (0.49–0.80) on the ROC curve (Fig. 4).

The radiomic model showed a sensitivity of 0.92 (95% CI 0.78–0.98) and a specificity of 0.56 (95% CI 0.37–0.74), computed using a probability threshold of 0.49. The ht-TKV model resulted in a sensitivity of 0.62 (95% CI 0.42–0.78) and a specificity of 0.72 (95% CI 0.53–0.87), considering a threshold of 0.48. With regards to the internal validation of the radiomic model the bootstrapped ‘average’ model confirmed the AUC value obtained during model fitting, while presenting a moderate decrease for sensitivity (0.80) and a slight increase for specificity (0.60).

## DISCUSSION

The major hallmark of ADPKD is the formation of multiple fluid-filled cysts in the kidneys, which subvert renal parenchyma and its function, leading to ESKD [14, 15]. The molecular driver for cyst enlargement is the production of pro-inflammatory mediators.

Fibrosis resulting from excessive accumulation of extracellular matrix proteins (ECM), and inflammation are among the most studied signatures of ADPKD [16, 17]. The link between cyst progression and injury has been suggested by Weimbs *et al*., who proposed a possible role for polycystin 1 (PC1) in sensing renal injury and leading to repair injured kidney tissue. In ADPKD, suppressed levels of PC1 eventually trigger proliferation, inflammatory derangement and cyst expansion [18]. Caroli *et al*. showed the histological fibrotic nature of specific areas of kidney parenchyma previously identified by means of contrast-enhanced CT and demonstrated a strong correlation of fibrosis with GFR measured at the time of CT imaging and the slope of GFR decline [19]. Similarly, the potential role of fibrosis as predictive factor for the onset of renal damage and for disease progression has been investigated and confirmed by Lai *et al*. on ADPKD kidneys studied by MRI [20].

ECM remodelling may be an attractive predictive biomarker for development of innovative models.

TKV predicts kidney function decline and is widely used to estimate risk of progression towards ESKD [21–25]. TKV can be calculated using either MRI, CT (both considered ‘gold standard’ techniques as being able to provide highly accurate measurements) or ultrasonography [26–28].

The MIC, which is based on age- and height-adjusted TKV, is an essential risk assessment tool for selecting ‘high-risk’ patients.

As technology in medicine is progressively playing an utterly relevant role, radiomics has recently become an attractive complementary tool in the context of evidence based clinical-decision support systems. In radiomics, digitally encrypted medical images holding information related to pathophysiology are transformed into vast arrays of quantitative features that can be used to identify prediction targets, such as clinical endpoints [8, 29, 30]. The field of application of radiomics in nephrology has been limited, so far, in developing reliable biomarkers for risk stratification and for prediction of outcomes to oncological therapies in neoplastic renal lesions [31].

The parenchyma of kidneys is the setting of molecular derangements which we aimed to explore by CT imaging and through a novel approach based on radiomics. Only few studies have lately focused on the ability of radiomics to predict kidney function in ADPKD. Cong *et al*. proposed a mixed clinical-radiomic model based on MR images that revealed being able to predict kidney function in ADPKD [32].

Kline *et al*. showed that specific texture features from kidney tissue extracted from MR images in addition to biomarkers such as ht-TKV can predict decline of renal function over time [33]. However, neither they internally validated their findings nor they assessed their model generalizability.

Xie *et al*. proposed a combined model based on renal parenchymal volume in ADPKD and radiomics features as an innovative tool for predicting renal function impairment through a prospective study based on CT scans [34]. Furthermore, recently, Kremer *et al*. demonstrated radiomic texture-based differences among risk-stratified MIC groups in ADPKD from MR images from both non-cystic and entire kidney parenchyma [35]. Li *et al*., in order to find a tool able to assist clinical decision-making, proposed a combination model of clinical factors and radiomics based on MR technique which showed effectiveness in predicting decline of kidney function [36].

Our hypothesis is that CT-based radiomics applied to kidney parenchyma as the background of inflammation and fibrosis could predict kidney function at baseline, on top of TKV, as well as kidney function decline over time. Differently from previous studies, we acknowledged rapid kidney function decline considering a minimum threshold of eGFR slope of 3 mL/min/1.73 m^2^/year and the radiomic model we built predicted it more efficiently compared with TKV. We extracted radiomic features from renal parenchyma and explored the most statistically significative ones for its potential to predict kidney function at baseline.

**Figure 5:**
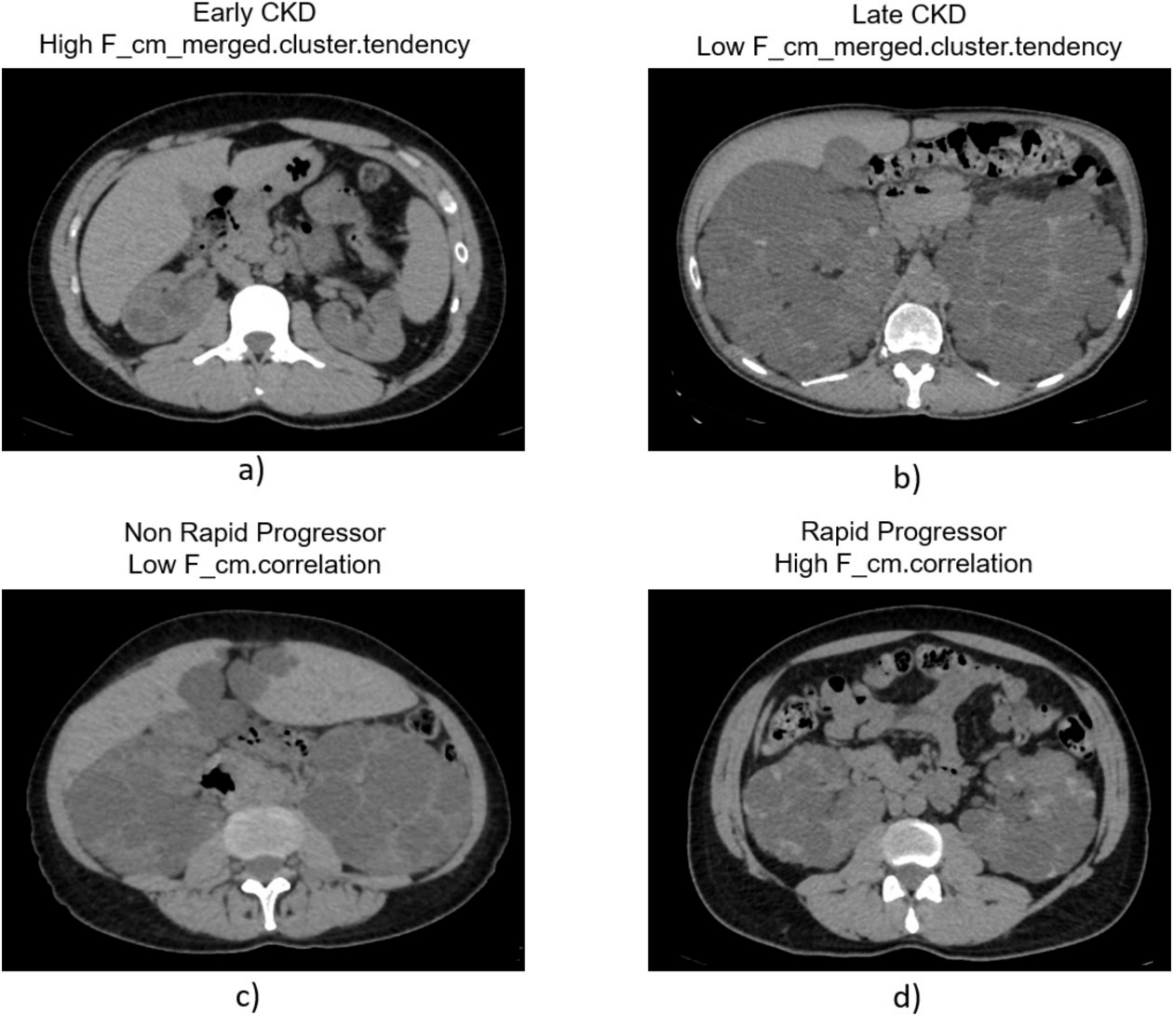
(**a**) Representative CT image from an early CKD patients corresponding to a high value of *F_cm_merged.clust.tend* due to more uniform intensity distributions. (**b**) Representative CT image from a late CKD patients corresponding to a low value of *F_cm_merged.clust.tend* due to greater textural complexity. (**c**) Representative CT image from non-rapid progressor patients corresponding to low value of *F_cm.correlation* due to a more homogeneous tissue. (**d**) Representative CT image from rapid progressor patients corresponding to high value of *F_cm.correlation* due to a more heterogeneous tissue.

The present study proposes a novel prediction model based on age and on a selected textural radiomic feature, namely the cluster tendency (*F_cm_merged.clust.tend*) which represents a measure of heterogeneity of kidney parenchyma by measuring groups of voxels with similar grey-level values. Homogeneous tissues exhibit a higher radiomic feature cluster tendency due to more uniform intensity distributions, while heterogeneous tissues have lower cluster tendency due to greater textural complexity. In our case, higher values for this feature were obtained for early CKD (≥60 mL/min/1.73 m^2^) compared with late CKD (<60 mL/min/1.73 m^2^) (Fig. 5a and b). The radiomic-age model based on CT images from polycystic kidneys, with an AUC of 0.91, sensitivity of 0.82 and specificity of 0.90, resulted the most effective in the prediction of baseline kidney function in our cohort.

Furthermore, we aimed to shed some light on the still unexplored ability of radiomics to predict kidney function slope over time among a limited patients’ cohort. Taking into account the above-mentioned threshold of eGFR slope of 3 mL/min/1.73 m^2^ to identify rapid progressors [5], the logistic regression model based on *F_cm.corr* showed an AUC 0.78 (95% CI 0.65–0.90) and predicted a steeper slope of kidney function over time for the rapid progressors with high sensitivity whereas, on the other hand, logistic regression model based on the ht-TKV presented a lower AUC and no statistically significant correlation was found between MIC classes based on ht-TKV among rapid vs non rapid progressors. This radiomic feature (‘correlation’) represents a measure of correlation of the pixel values characterizing texture heterogeneity of kidney parenchyma. The value of this feature is typically higher for heterogeneous tissues compared with homogeneous tissues since intensity values fluctuate more in heterogeneous tissues: there is a stronger relationship between different grey levels, resulting in a higher correlation value. In our cohort, higher values of this feature were observed for the rapid progressors compared with the non-rapid progressors. The imbalance performance of the developed radiomic model may be improved in future studies including large sample sizes which will allow to use multiple radiomic features in combination with clinical variables.

The limitations of this pilot study corresponding to a TRIPOD (Transparent reporting of a multivariable prediction model for individual prognosis or diagnosis) level of 2A are the limited number of cases and the use of retrospective data from a single centre [12]. Furthermore, we did not assess inter-observer reproducibility of CT segmentation and did not perform image discretization prior to feature extraction, thus robustness of radiomic features in this context may be evaluated in future studies.

This is among the first studies that aimed to investigate the potential ability of radiomics to discriminate eGFR at baseline and to predict rapid kidney function impairment over time in a clinical setting. This could represent a groundbreaking approach in the stratification of patients’ risk of progression towards ESKD and in the identification of patients who may benefit from disease-modifying therapies. In order to confirm the supporting role of radiomics to clinical decisions, further studies with larger cohorts and external validation are needed.

## Data Availability

The data underlying this article will be shared on reasonable request to the corresponding author.
